# Recent Progress in the Drug Development Targeting SARS-CoV-2 Main Protease as Treatment for COVID-19

**DOI:** 10.3389/fmolb.2020.616341

**Published:** 2020-12-04

**Authors:** Wen Cui, Kailin Yang, Haitao Yang

**Affiliations:** ^1^Institute of Life Sciences, Chongqing Medical University, Chongqing, China; ^2^School of Life Science and Technology, Shanghai Institute for Advanced Immunochemical Studies, ShanghaiTech University, Shanghai, China; ^3^Taussig Cancer Center, Cleveland Clinic, Cleveland, OH, United States; ^4^Tianjin International Joint Academy of Biomedicine, Tianjin, China

**Keywords:** SARS-CoV-2, COVID-19, main protease, repurposed drugs, *ab initio* drug design

## Abstract

The sudden outbreak of 2019 novel coronavirus (2019-nCoV, later named SARS-CoV-2) rapidly turned into an unprecedented pandemic of coronavirus disease 2019 (COVID-19). This global healthcare emergency marked the third occurrence of a deadly coronavirus (CoV) into the human society after entering the new millennium, which overwhelmed the worldwide healthcare system and affected the global economy. However, therapeutic options for COVID-19 are still very limited. Developing drugs targeting vital proteins in viral life cycle is a feasible approach to overcome this dilemma. Main protease (M^pro^) plays a dominant role in processing CoV-encoded polyproteins which mediate the assembly of replication-transcription machinery and is thus recognized as an ideal antiviral target. Here we summarize the recent progress in the discovery of anti-SARS-CoV-2 agents against M^pro^. Combining structural study, virtual screen, and experimental screen, numerous therapeutic candidates including repurposed drugs and *ab initio* designed compounds have been proposed. Such collaborative effort from the scientific community would accelerate the pace of developing efficacious treatment for COVID-19.

## Introduction

Our world is currently suffering from an unprecedented and emergent pandemic of coronavirus disease 2019 (COVID-19), which causes symptoms including fever, cough, pneumonia, nausea, and respiratory failure (Sohrabi et al., 2020). As of October 9th, a total of 36,361,054 cases of COVID-19 occurring in at least 235 countries and territories were reported, with ~2.9% of fatality rate (1,056,186 deaths). The etiology of COVID-19 was identified as severe acute respiratory syndrome coronavirus 2 (SARS-CoV-2), which spread through the mucus membrane of both upper and lower respiratory tracts to infect other cells within the body, whilst inducing a cytokine storm and generating a series of immune responses (Blanco-Melo et al., 2020). Phylogenetic analysis revealed that SARS-CoV-2 is classified into the genus lineage B *Betacoronavirus*, including severe acute respiratory syndrome coronavirus (SARS-CoV) and Middle East respiratory syndrome coronavirus (MERS-CoV). Like SARS-CoV and MERS-CoV, SARS-CoV-2 might also be transmitted to human from animal reservoirs, such as bats and pangolins (Andersen et al., 2020; Zhou et al., 2020).

Coronavirus (CoV) is an enveloped, single-stranded positive-sense RNA virus with the largest genome size ranging from approximate 26 to 32-kilobases found to date (Chen et al., 2020). Approximately two-thirds of the 5′ genome encodes two overlapping polyproteins, pp1a and pp1ab, which are essential for viral replication and transcription. The 3′ terminus encodes a canonical set of four structural proteins for coronavirus: nucleocapsid (N), spike protein (S), membrane protein (M), and envelope protein (E), which are responsible for virion assembly and suppression of host immune response. In the life cycle of CoV infection, it mainly uses spike proteins to bind to their receptors for attachment onto the host cell membrane. Then, CoV fuses with host cellular membrane and releases its genomic RNA. Subsequently, the two polyproteins are expressed through hijacking host ribosomes, which are further processed by two viral proteases, papain-like protease and main protease (M^pro^), into 16 mature non-structural proteins (nsps). These nsps, including helicase, RNA-dependent RNA polymerase (RdRp), and methyltransferase, can then assemble into the replication-transcription complex and initiate viral RNA replication and translation (Thiel et al., 2003). The newly produced viral RNA and proteins are then packaged into mature progeny virions, which are subsequently released through exocytosis to infect other healthy cells.

Numerous therapeutics have been reported to effectively inhibit SARS-CoV-2 replication since the outbreak of the pandemic in late 2019 (Tu et al., 2020). They mainly target the essential proteins in the life cycle of the virus. Remdesivir is the most promising drug up to now, which interferes the viral genome replication by targeting RdRp (Warren et al., 2016). Remdesivir resembles the structure of adenosine, enabling it to incorporate into nascent viral RNA and result in premature termination of the viral RNA chain. In the National Institutes of Health (NIH)-sponsored Adaptive COVID-19 Treatment Trial (ACTT), Gilead Sciences-sponsored treatment trial, and another ongoing phase 3, randomized, open-label trial (GS-US-540-5773), remdesivir showed promising clinical efficacy (Beigel et al., 2020; Goldman et al., 2020; Olender et al., 2020). Another recently reported potential drug is APN01, which could inhibit SARS-CoV-2 replication in cellular and embryonic stem cell-derived organoids. It is a soluble recombinant human angiotensin-converting enzyme 2 (ACE2), and could prevent the activation of cellular ACE2, which is the host receptor for viral S protein (Monteil et al., 2020). Through interfering with the maturation of nsps, numerous drug candidates which inhibit the M^pro^ activity have been discovered through pharmaceutical screening, such as ebselen, disulfiram, carmofur, α-ketoamides, and peptidomimetic aldehydes 11a/11b (Dai et al., 2020; Jin et al., 2020a,b; Zhang et al., 2020b). All these agents can be divided into two categories. The first category is repurposed drugs (Pushpakom et al., 2019) which originally designed for other diseases. The other is the *ab initio* designed drugs based on the structure characterization. Although several existing antiviral drugs have shown good results in clinical trials, continued efforts to discover new drugs that efficiently treat COVID-19 are urgently needed to address the ongoing pandemic. Here, we mainly focus on M^pro^ of SARS-CoV-2 and summarize the recent important progresses in its drugs targeting, which may facilitate the development of effective therapeutic agents to fight against this novel pathogen.

## Main Protease

**Figure 1:**
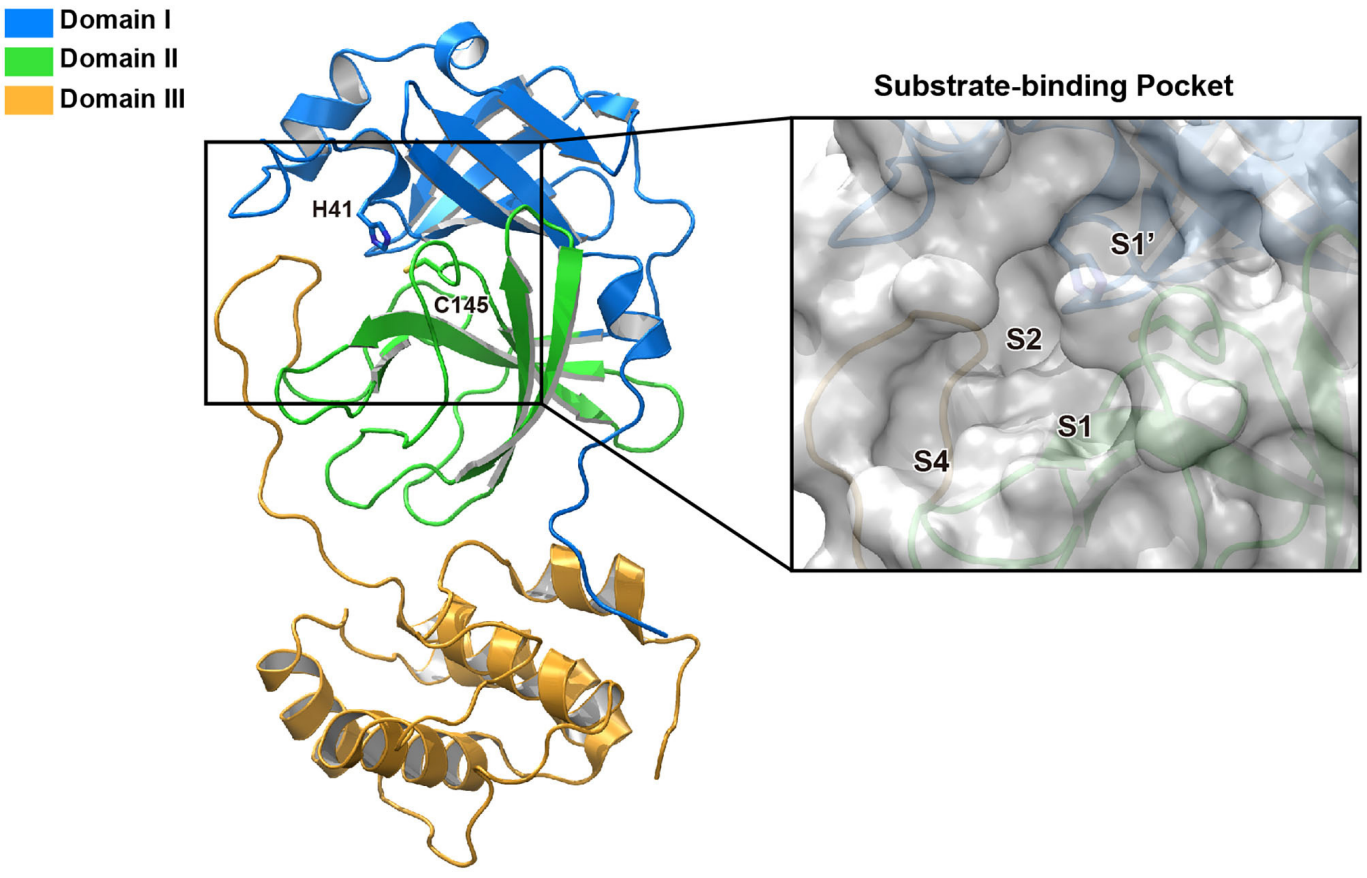
The structure of SARS-CoV-2 M^pro^. Ribbon representation of SARS-CoV-2 M^pro^ (PDB entry: 6LU7). The substrate-binding pocket with transparent surface is shown in the inset with subsites labeled.

M^pro^, also termed 3CL protease, is a 33.8-kDa cysteine protease which mediates the maturation of functional polypeptides involved in the assembly of replication-transcription machinery (Wang H. et al., 2016). M^pro^ digests the polyprotein at no less than 11 conserved sites, starting with the autolytic cleavage of this enzyme itself from pp1a and pp1ab. In addition, M^pro^ has no human homolog and is highly conserved among all CoVs (Yang et al., 2006). These above features make it an attractive drug target against CoVs. After the outbreak of COVID-19, the crystal structure of SARS-CoV-2 M^pro^ was rapidly determined (Jin et al., 2020a), which greatly facilitated its mechanistic study and inhibitor development. Structure of M^pro^ from SARS-CoV-2 revealed a three-domain (domains I to III) architecture which is conserved among CoVs (Figure 1). In the cleft between domains I and II, it features a non-canonical Cys-His dyad as the catalytic site. The cysteine residue of the Cys-His dyad undergoes nucleophilic attack on the reactive atom of the substrate, while the histidine residue helps to stabilize the intermediate state. Around this dyad, M^pro^ forms a conserved binding pocket which is composed of four subsites (S1′, S1, S2, and S4) well accommodating the substrate (Xue et al., 2008). The amino acid residues of the substrate are usually numbered as (P4-P3-P2-P1↓P1′-P2′) from the N terminus to C terminus around the cleavage site, and the typical sequence recognized and cleaved by M^pro^ is Leu-Gln↓ (Ser, Ala, Gly) in the polyproteins (Yang et al., 2005). Remarkably, the Gln residue and hydrophobic residue with long side chain are almost always required in the P1 position and P2 position, which correspond to the conserved and large subsites of S1 and S2, respectively.

## Inhibitors Targeting SARS-CoV-2 M^Pro^

Lopinavir and ritonavir were the first drugs used in clinical trials to treat COVID-19 targeting M^pro^ (Cao et al., 2020). They are inhibitors to human immunodeficiency virus (HIV) aspartyl protease, which is encoded by the *pol* gene of HIV and cleaves the precursor polypeptides in HIV (Walmsley et al., 2002). The combination of lopinavir and ritonavir are commonly used as a therapeutic regimen for patients with HIV infection (Cvetkovic and Goa, 2003). Lopinavir was previously shown to inhibit M^pro^ of SARS-CoV *in vitro* (Wu et al., 2004), and further studies demonstrated promising antiviral capacity of lopinavir/ritonavir against SARS-CoV and MERS-CoV (Chan et al., 2003, 2015). Since M^pro^ is highly conserved between SARS-CoV-2 and SARS-CoV, lopinavir/ritonavir was proposed as a potential therapeutic option during the early phase of COVID-19 pandemic (Baden and Rubin, 2020; Liu and Wang, 2020; Wang, 2020). Lopinavir/ritonavir combination was evaluated in a clinical trial against COVID-19 in patients with mild or moderate COVID-19 (NCT04252885), however it showed little improvement of clinical outcome (Li et al., 2020). In another trial performed on hospitalized patients with severe COVID-19 (ChiCTR2000029308), no benefit in the time to clinical improvement was observed in the lopinavir/ritonavir arm when compared to patients receiving standard of care (Cao et al., 2020).

**Figure 2:**
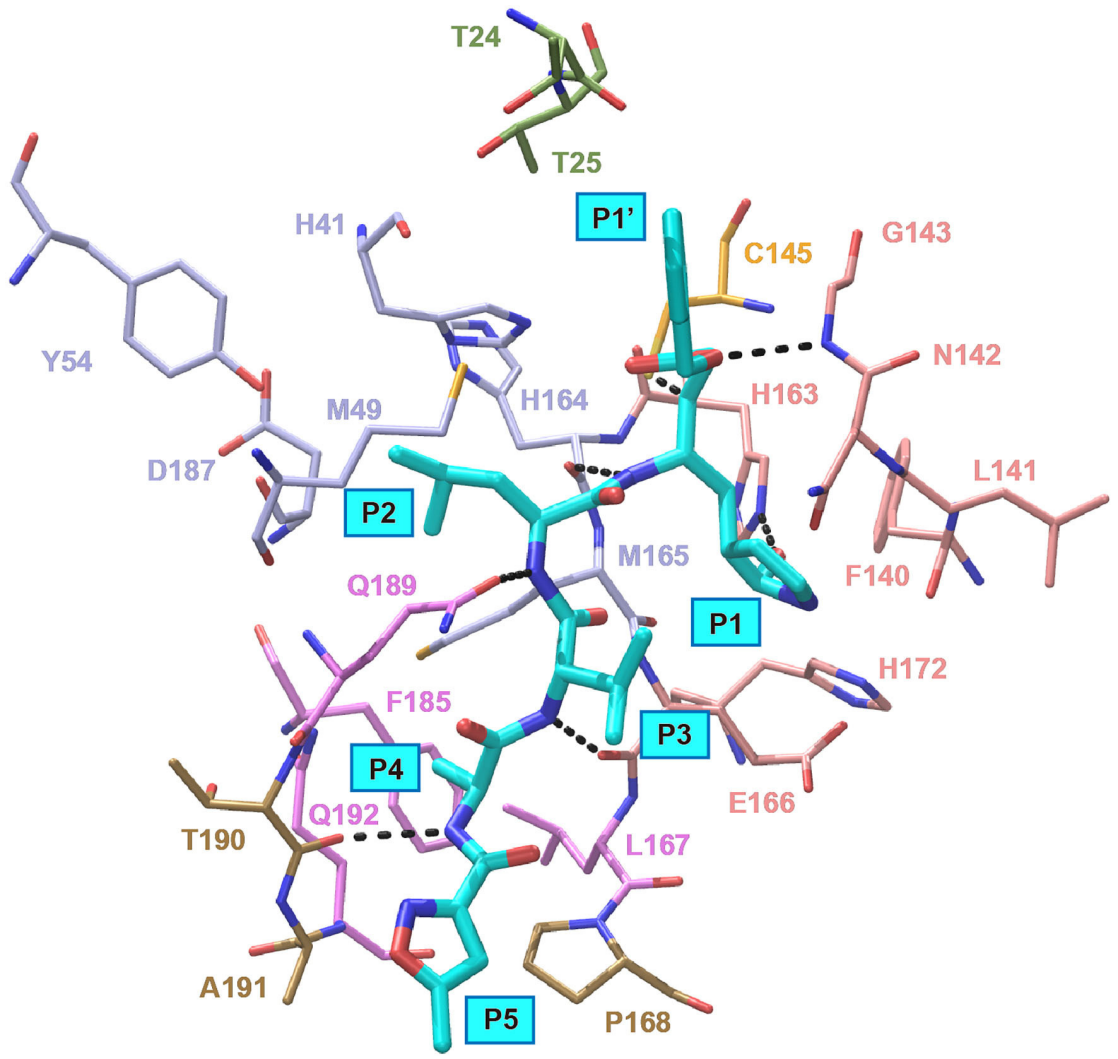
Interaction pattern between N3 and SARS-CoV-2 M^pro^. N3 is shown as cyan sticks, the residues interacting with P1′ are shown as green sticks, the residues forming the S1 site are shown as salmon sticks, the residues forming the S2 site are shown as light blue sticks, the residues forming the S4 site are shown as violet sticks and the residues interacting with P5 are shown as sand sticks. Intermolecular hydrogen bonds are shown as dashed lines.

**Figure 3:**
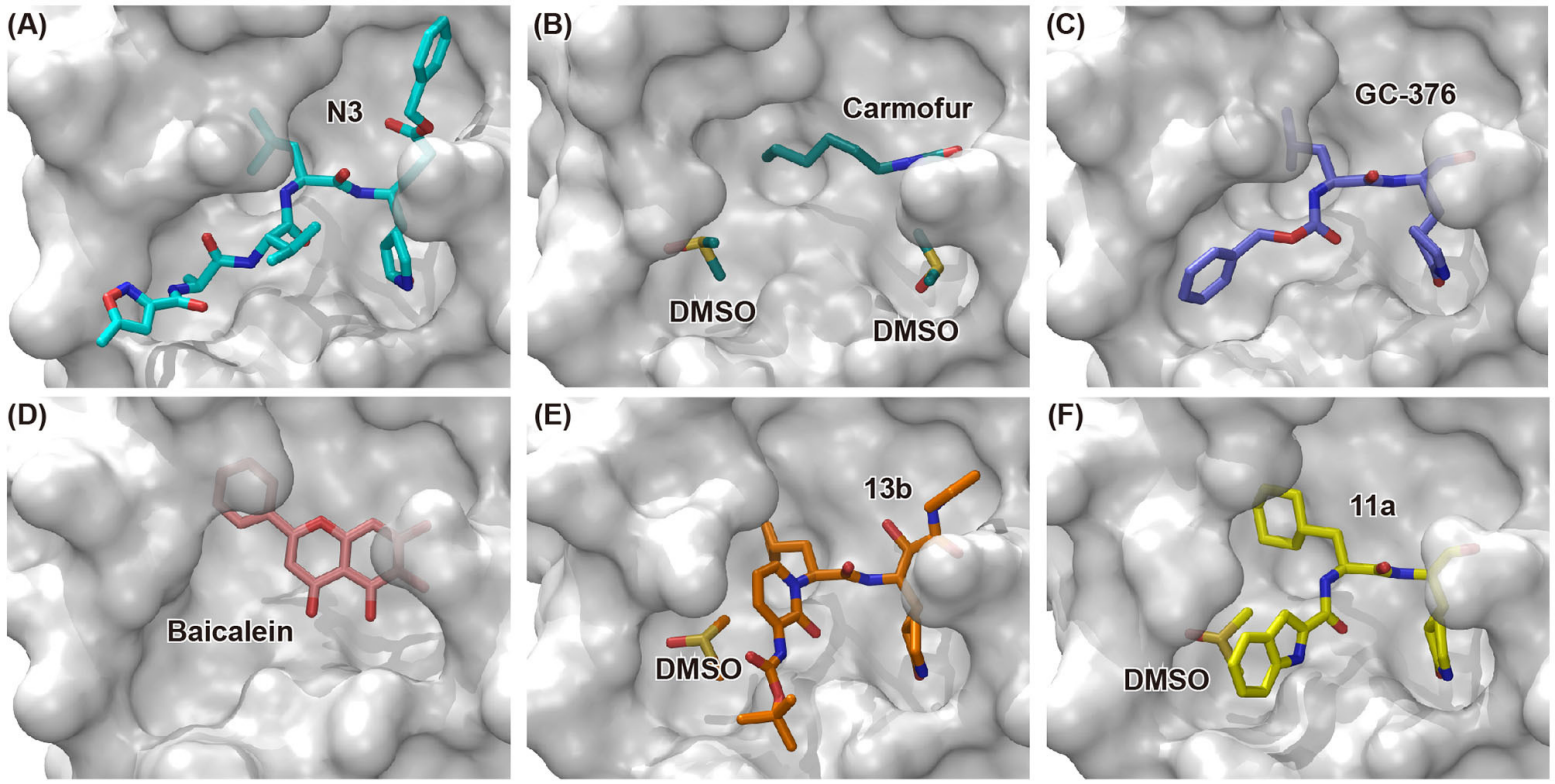
Binding modes of newly identified inhibitors in complex with SARS-CoV-2 M^pro^. Inhibitors and co-factors are shown in stick: **(A)** N3 (PDB entry: 6LU7) (Jin et al., 2020a), **(B)** carmofur (PDB entry: 7BUY) (Jin et al., 2020b), **(C)** GC-376 (PDB entry: 6WTT) (Ma et al., 2020), **(D)** baicalein (PDB entry: 6M2N) (Su et al., 2020), **(E)** 13b (PDB entry: 6Y2F) (Zhang et al., 2020b), and **(F)** 11a (PDB entry: 6LZE) (Dai et al., 2020). The catalytic active site of SARS-CoV-2 M^pro^ is shown in surface mode.

In an effort to discover more potent inhibitors, Jin et al. reported several repurposed drugs targeting M^pro^ based on inhibitory mechanism of N3 (Table 1) (Jin et al., 2020a). N3 is a Michael acceptor-based peptidomimetic inhibitor (Yang et al., 2005), which was originally designed to treat infectious disease caused by other CoVs (Wang et al., 2016a,b, 2017). It utilizes a vinyl group to inhibit the catalytic process via covalent bond to the cystine of the catalytic dyad. In addition, it harbors a lactam ring, an aliphatic isobutyl group, and a methyl group as the side chain of P1, P2, and P4, respectively, which well fit the subsites S1, S2, and S4. Unsurprisingly, N3 was also found to exhibit potent inhibition to SARS-CoV-2 M^pro^. By determining the crystal structure of SARS-CoV-2 M^pro^ in complex with N3, the inhibitory mechanism was identified: besides the covalent bond formed between the Sγ atom of C145 and the Cβ of the vinyl group, the P1 side chain protrudes into the S1 subsite, which is composed of F140, L141, N142, G143, E166, H163, and H172. The S2 subsite which consists of H41, M49, Y54, H164, M165, and D187, deeply buries the P2 side chain. The shallow S4 subsite formed by L167, F185, Q189, and Q192, accommodates the small side chain of P4. P1' and P5 make van der Waals contacts with P168, T190, A191 and T24, T25, respectively. In addition, multiple hydrogen bonds are formed between N3 and the residues in the binding pocket, which lock the inhibitor inside (Figure 2). Because N3 has substituents spanning all substrate binding subsites, the recognition mechanism between N3 and M^pro^ should represent the canonical pattern for inhibitors and M^pro^ (Figure 3A).

**Table 1:** Antiviral activity of newly discovered hits against SARS-CoV-2.

**Compounds**	**SARS-CoV-2 M^**pro**^ inhibition (μM)**	**SARS-CoV-2 antiviral activity (μM)**	**Derived from**
N3			
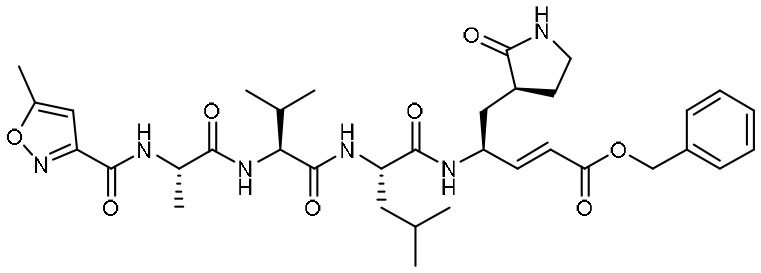	*k_*obs*_*/[I] = 11,300 M^−1^ s^−1^	EC_50_ = 16.77	*ab initio* designed drug
Ebselen			
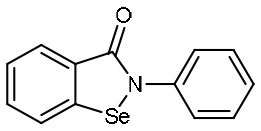	IC_50_ = 0.67	EC_50_ = 4.67	In clinical trial
Carmofur			
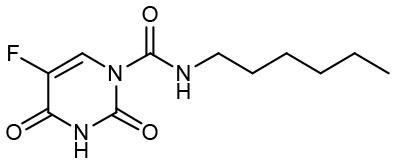	IC_50_ = 1.82	EC_50_ = 24.87	FDA-approved drug
Boceprevir			
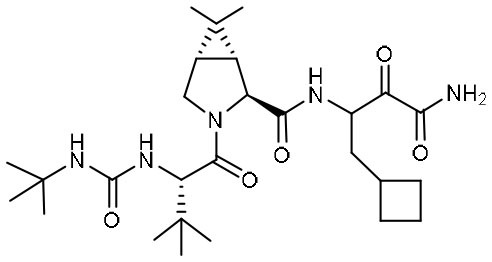	IC_50_ = 4.13	EC_50_ = 1.9	FDA-approved drug
GC-376			
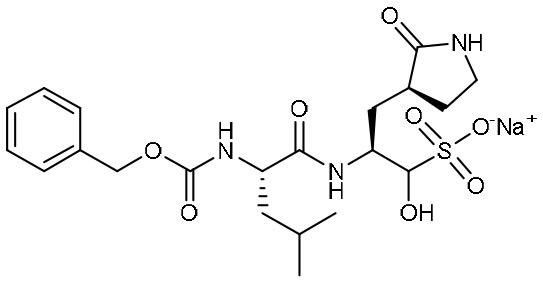	IC_50_ = 0.03	EC_50_ = 2.07	Preclinical
Calpain inhibitor II			
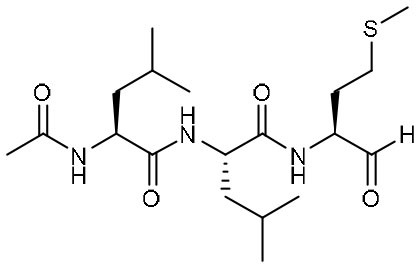	IC_50_ = 0.97	EC_50_ = 0.49	Preclinical
Calpain inhibitor XII			
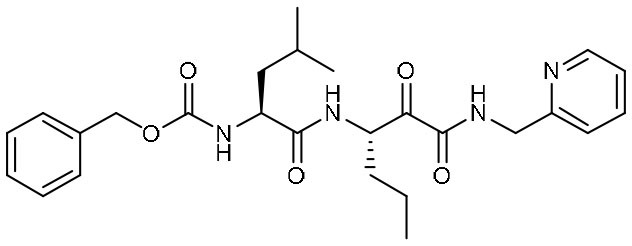	IC_50_ = 0.45	EC_50_ = 3.37	Preclinical
Baicalein			
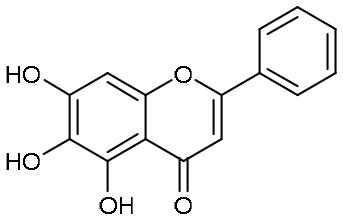	IC_50_ = 0.94	EC_50_ = 1.69	Chinese medicine
13b			
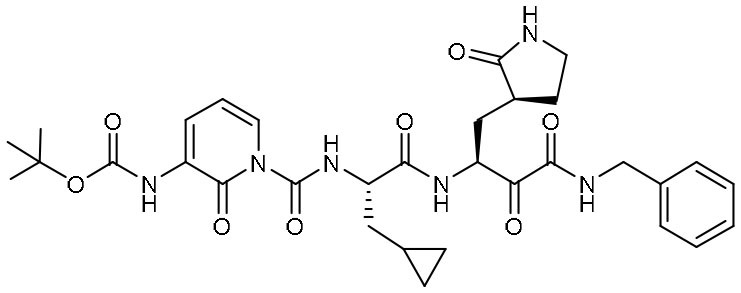	IC_50_ = 0.67	EC_50_ = 4~5	*ab initio* designed drug
11a			
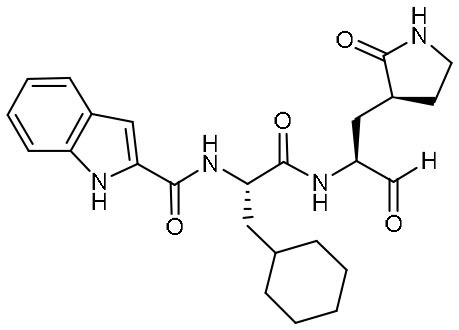	IC_50_ = 0.05	EC_50_ = 0.53	*ab initio* designed drug
11b			
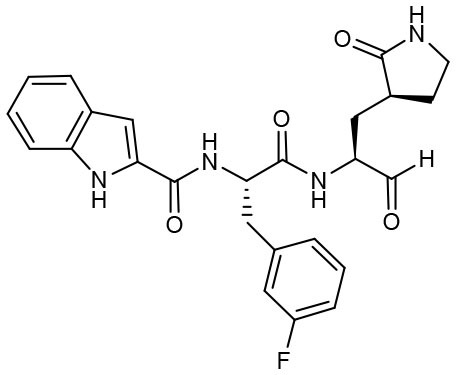	IC_50_ = 0.04	EC_50_ = 0.72	*ab initio* designed drug
6e			
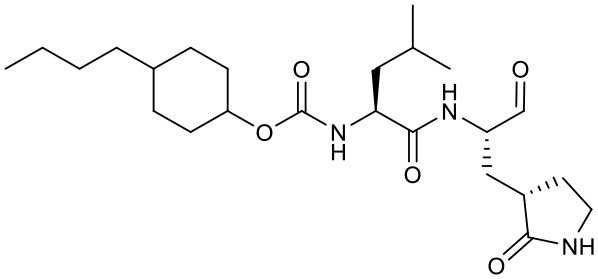	IC_50_ = 0.17	EC_50_ = 0.15	*ab initio* designed drug

Based upon this mechanism, over 10,000 compounds from a library consisted of approved drugs, drug candidates in clinical trials, and other pharmacologically active compounds were screened, and several potent inhibitors to SARS-CoV-2 were discovered, such as disulfiram, carmofur, Ebselen, shikonin, tideglusib, PX-12, and TDZD-8 (Jin et al., 2020a). Among these candidates, ebselen is an organoselenium compound which has been investigated for the treatment of multiple diseases (Table 1), such as bipolar disorders and hearing loss, and its safety in humans has been evaluated in a number of clinical trials (Kil et al., 2007). Ebselen displayed strong inhibition of M^pro^ activity against SARS-CoV-2 with a half maximal inhibitory concentration (IC_50_) of 0.67 μM and a half maximal effective concentration (EC_50_) of 4.67 μM, respectively. It also showed the capacity to penetrate cellular membrane to access their targets and extremely low cytotoxicity, with half maximal lethal dose (LD_50_) in rats > 4,600 mg/kg. Disulfiram is an FDA-approved drug for the treatment of alcohol dependence (Garbutt et al., 1999). It was also investigated as a repurposed drug for the treatment of cancers during the past few years (Skrott et al., 2017). It exhibited strong inhibition of M^pro^ activity against SARS-CoV-2 with an IC_50_ of 9.35 μM. Remarkably, both ebselen and disulfiram are currently under evaluation in phase II clinical trials for the treatment of COVID-19 (NCT04484025 and NCT04485130). Taken together, these data strongly support the clinical potential of these drugs to treat COVID-19.

Carmofur is an approved antineoplastic drug, derived from 5-fluorouracil (5-FU), and has been investigated in curing breast, gastric, bladder, and colorectal cancers (Sakamoto et al., 2005; Dementiev et al., 2019). It was also reported as a novel therapeutic agent for acute lung injury (ALI) (Wu et al., 2019). Carmofur displayed inhibition of M^pro^ activity and against SARS-CoV-2 with an IC_50_ of 1.82 μM and an EC_50_ values of 24.87 μM (Table 1). Complex structure of SARS-CoV-2 M^pro^ with carmofur showed that carmofur covalently binds to the catalytic cysteine via an electrophilic carbonyl reactive group (Figure 3B) (Jin et al., 2020b). Its fatty acid tail occupies the hydrophobic S2 subsite of M^pro^ whilst its 5-fluorouracil head is cleaved off (Jin et al., 2020b). These findings make carmofur a novel and promising lead compound for the development of antivirals to target COVID-19.

Using experimental screening method, Ma et al. discovered several drug candidates targeting M^pro^ (Ma et al., 2020). Utilizing FRET-based enzymatic assay, repurposed drugs including boceprevir, GC-376, and calpain inhibitors II and XII, were found to exhibit potent inhibition to M^pro^ with IC_50_ values of 4.13, 0.03, 0.97 and 0.45 μM, respectively (Table 1). In further *in vitro* antiviral activity test, these repurposed drugs also showed strong inhibition to SARS-CoV-2 viral replication in cell culture with EC_50_ values ranging from 0.49 to 3.37 μM. Among these compounds, boceprevir is an FDA-approved drug for hepatitis C virus (HCV) (Yang et al., 2003), which has been extensively evaluated in its safety and would speed up its entry into clinical studies toward COVID-19. GC-376 is an investigational veterinary drug, which has promising *in vivo* efficacy in treating feline infectious peritonitis (FIP) in cats and has favorable pharmacokinetic properties (Pedersen et al., 2018). Crystal structure of SARS-CoV-2 M^pro^ in complex with GC-376 showed that the aldehyde bisulfite warhead forms a covalent bond to the cysteine residue of the catalytic dyad (Figure 3C). The γ-lactam ring in P1, the isobutyl moiety in P2, and a phenylmethyl ester in P4 exhibit optimal complementarity with subsites S1, S2, and S4, respectively. Calpain inhibitors II and XII were initially identified as inhibitors to calpain, which is a host enzyme required for the proteolytic processing of the S protein of CoV (Barnard et al., 2004; Ono et al., 2016). Having the capacity to target M^pro^ and calpain simultaneously, calpain inhibitors II and XII would exhibit more potential for effective inhibition to virus.

In another search for therapeutic agents, Su et al. found that ingredients from Chinese traditional medicines also exhibit inhibitory efficacy against SARS-CoV-2 via targeting M^pro^ (Su et al., 2020). Baicalin and baicalein are natural products derived from *S. baicalensis*, which are frequently used for prophylaxis and treatment of hepatitis and respiratory disorders (Dinda et al., 2017). In this study, both products displayed significant inhibition against M^pro^ with an IC_50_ of 6.41 and 0.94 μM, respectively (Table 1). Cell-based antiviral activities of baicalin and baicalein were superior to many of the reported compounds with an EC_50_ of 10.27 and 1.69 μM, respectively. The determined structure of M^pro^ in complex with baicalein showed a novel non-covalent inhibition model (Figure 3D). The phenyl ring with three hydroxyl groups of baicalein formed π-S and π-π interactions with the catalytic dyad C145 and H41, while the other hydroxyl groups made hydrogen bonds to multiple residues in the S1 subsite. The free phenyl ring occupied the S2 subsite. The baicalein molecule acted as a lid on the catalytic site to block the entry of peptide substrate. The unique mode of action, potent antiviral activities *in vitro*, and the favorable safety data from clinical trials, make baicalein a promising agent for clinical trials and a novel drug lead targeting M^pro^.

Peptidomimetic α-ketoamides are broad-spectrum inhibitors of the M^pro^ of *betacornaviruses* and *alphacoronaviruses* as well as the 3C protease of enteroviruses, which were previously designed according to a structure-based approach (Zhang et al., 2020a). An α-keto group was employed to covalently inhibit the nucleophilic attack of the catalytic cysteine residue onto the substrate. In addition, these inhibitors were equipped with a γ-lactam and hydrophobic group at P1 and P2 respectively, to occupy the characteristic subsites S1 and S2 and enhance inhibitory efficacy. In a previous study, 11r was identified as the best inhibitor among them against CoVs (Zhang et al., 2020a). Recently, through modifications to 11r, Zhang et al. proposed a new inhibitor named 13b with improved inhibitory effect: the P3-P2 amide bond was embedded within a pyridone ring (Figure 3E), which prevented it from cleavage by cellular proteases and enhanced its half-life (T_1/2_) in plasma (Zhang et al., 2020b). In addition, the P2 cyclohexyl moiety was replaced by the smaller cyclopropyl, which strengthened its specific antiviral activity against *betacoronaviruses*. This newly generated compound 13b inhibited the SARS-CoV-2 M^pro^ with IC_50_ = 0.67 μM and viral RNA replication with EC_50_ = 4~5 μM (Table 1). In mice, 13b also displayed 3-times plasma half-life compared to 11r. The pharmacokinetic characterization of 13b revealed a pronounced pulmonary tropism and suitability for administration through inhalation. All these characteristics make 13b a potential therapeutic agent toward COVID-19.

Targeting M^pro^ of SARS-CoV-2, Dai et al. presented peptidomimetic aldehydes as antiviral drug candidates within structure-based *ab initio* drug design (Ramajayam et al., 2011; Dai et al., 2020). They synthesized two compounds, named as 11a and 11b, which use peptidomimetic aldehydes as their framework and differ in the P2 group (Table 1). Both compounds exhibited excellent inhibitory activity with an IC_50_ value of 0.05 and 0.04 μM, respectively. They also demonstrated potent efficacy against SARS-CoV-2 infection in a cell-based assay with an EC_50_ value of 0.53 and 0.72 μM, respectively. Additionally, they showed good pharmacokinetic properties in animal model: as for 11a, when given intraperitoneally and intravenously, the half-life is 4.27 and 4.41 h, respectively. The bioavailability of intraperitoneal route is 87.8%. For 11b, when given intraperitoneally the T_1/2_ is 5.21 h, with the bioavailability of more than 80%. 11a also exhibited low toxicity with LD_25_ = 60 mg/kg in SD rats. The crystal structures of SARS-CoV-2 M^pro^ in complex with 11a and 11b showed that their aldehyde groups are bound covalently to catalytic cysteine to inhibit M^pro^ activity (Figure 3F). Altogether, 11a and 11b are efficacious drug leads with clinical potential that merit further study.

Most recently, Rathnayake et al. reported that a series of inhibitors of M^pro^, which were designed and optimized based on the structure, are effective against multiple human CoVs including MERS-CoV, SARS-CoV, and SARS-CoV-2 (Rathnayake et al., 2020). These inhibitors are derived from modification of their previously designed dipeptidyl compounds (Prior et al., 2013). These inhibitors utilized aldehyde group and γ-lactam ring for covalent bond to catalytic cysteine and P1 side chain and differ in P2 and P4 side chain. Among them, 6j was found to show the most potent inhibition to MERS-CoV M^pro^ and the most potent antiviral activity against MERS-CoV with an IC_50_ of 0.08 μM and an EC_50_ of 0.04 μM. Meanwhile, 6e showed the most potent inhibition to SARS-CoV-2 M^pro^ and the most potent antiviral activity against SARS-CoV-2 with an IC_50_ of 0.17 μM and an EC_50_ of 0.15 μM (Table 1). In cultured primary human airway epithelial cells from patients infected with SARS-CoV-2, 6e and 6j exhibited evident inhibition to virus replication. Using mouse model of MERS-CoV infection, administration of 6j resulted in increased survival rate, faster recovery of body weight, reduced lung viral titers, and reduced lung histopathology. All these results laid the foundation for the development of such dipeptidyl compound series into potential broad-spectrum antiviral drugs against human CoVs.

## Virtual Screening Targeting M^Pro^

With the precisely defined structure and substrate-binding mode, virtual screening was employed in several studies to discover drug candidates targeting M^pro^. Ton et al. utilized Deep Docking methodology in conjunction with Glide to estimate the inhibition effect of 1.3 billion compounds from ZNIC15 library and identified 1,000 potential compounds which demonstrated superior scores of docking into the active site of SARS-CoV-2 M^pro^ (Ton et al., 2020). Fischer et al. launched a computational search for M^pro^ inhibitors: through a series of screening steps including shape screen, smina docking, Glide docking, clustering, pharmacokinetic descriptors, MD simulations and toxicity assessment, they finally obtained 13 hits out of 606 million compounds from the ZINC database (Fischer et al., 2020). In another report, Joshi et al. discovered ligands targeting M^pro^ in a multi-target-directed approach: they primarily identified nine natural molecules, such as myricitrin, taiwanhomoflavone A, and lactucopicrin 15-oxalate from ~7100 molecules (Joshi et al., 2020). But additional docking analysis showed that these compounds also exhibited potent binding to other targets including ACE2 and RdRp. Since these inhibitors have the capacity to target multiple essential proteins simultaneously, they merit further pharmaceutical investigation regarding their antiviral efficacy.

Besides the experimental screening of drug candidates, computational method was also utilized to design novel drugs against M^pro^. Choudhury et al. first analyzed the binding of 191,678 molecular fragments to different constituent subsites of the SARS-CoV-2 M^pro^ (Choudhury, 2020). Then the fragments with high affinity to adjacent subsites were selected and tailored into new molecules. Through *in silico* evaluation, 17 of these molecules were identified as showing promising binding capacity (Choudhury, 2020). It is noteworthy that there are numerous other reports on the virtual screening for drug candidates targeting M^pro^, and hundreds of potential therapeutic agents have been discovered (Aanouz et al., 2020; Enmozhi et al., 2020; Gupta et al., 2020; Islam et al., 2020; Khan et al., 2020; Peele et al., 2020). Though these findings have not been corroborated with experimental validation, they acted to accelerate the pace of discovery for suitable drug candidates against COVID-19.

## Conclusions

After SARS-CoV and MERS-CoV, SARS-CoV-2 is now causing a pandemic of infectious respiratory disease, with a much wider and more significant impact on global healthcare and economy (Galea and Abdalla, 2020; Khullar et al., 2020). Facing with this severe and urgent situation, the sprint to find effective treatments has been dramatically accelerated. One of the potential treatment strategies is the discovery of drugs by targeting essential proteins in viral life cycle. M^pro^ become an attractive drug target, since it plays a pivotal role in mediating viral replication and transcription (Yang et al., 2006). Via virtual and experimental screenings, a series of drug candidates have been reported to date. Some of these agents were repurposed drugs, which previously designed for other applications with approved druggability (Jin et al., 2020a,b; Ma et al., 2020; Su et al., 2020). They showed good performance in *in vitro* anti-SARS-CoV-2 study and could rapidly enter further clinical trials. Other agents were *ab initio* designed drugs based on 3 dimensional structure of M^pro^ (Dai et al., 2020; Rathnayake et al., 2020; Zhang et al., 2020b), and these compounds demonstrated the advantage of more potent inhibition and specificity toward M^pro^.

With the aid of structural study, more accurate inhibitory modes were identified, which would provide unique insights into the further optimization of drug leads. It is worth mentioning that, in search for drug candidate, a strategy which combines structure-based *ab initio* drug design, virtual screening and experimental screen has exhibited a promising prospect (Jin et al., 2020a). Such strategy may facilitate the preparation of global biomedical community in advance for any future pandemic caused by emerging deadly virus.

## Author Contributions

HY designed the study. WC and KY wrote the manuscript. HY gave constructive comments on the manuscript and approved the manuscript before submission. All authors contributed to the article and approved the submitted version.

## Conflict of Interest

The authors declare that the research was conducted in the absence of any commercial or financial relationships that could be construed as a potential conflict of interest.

## Abbreviations

2019-nCoV: 2019 novel coronavirus
CoV: coronavirus
M^pro^: Main protease
COVID-19: coronavirus disease 2019
SARS-CoV-2: severe acute respiratory syndrome coronavirus 2
MERS-CoV: Middle East respiratory syndrome coronavirus
SARS-CoV: severe acute respiratory syndrome coronavirus
nsps: non-structural proteins
RdRp: RNA-dependent RNA polymerase
NIH: National Institutes of Health
ACTT: Adaptive COVID-19 Treatment Trial
ACE2: Angiotensin-converting Enzyme 2
HIV: human immunodeficiency virus
EC_50_: half maximal effective concentration
IC_50_: half maximal inhibitory concentration
LD_50_: lethal dose, 50%
5-FU: 5-fluorouracil
ALI: acute lung injury
HCV: hepatitis C virus
FIP: Feline Infectious Peritonitis
T_1/2_: half-life
LD_25_: lethal dose, 25%
MD: molecular dynamics.
